# Prevalence and antimicrobial susceptibility of *Vibrio parahaemolyticu*s isolated from retail shrimps in Malaysia

**DOI:** 10.3389/fmicb.2015.00033

**Published:** 2015-01-30

**Authors:** Vengadesh Letchumanan, Wai-Fong Yin, Learn-Han Lee, Kok-Gan Chan

**Affiliations:** ^1^Division of Genetics and Molecular Biology, Institute of Biological Sciences, Faculty of Science, University of MalayaKuala Lumpur, Malaysia; ^2^Jeffrey Cheah School of Medicine and Health Sciences, Monash University MalaysiaBandar Sunway, Malaysia

**Keywords:** *Vibrio parahaemolyticus*, food borne, virulence factors, antimicrobial, MAR index

## Abstract

*Vibrio parahaemolyticus* is a marine and estuarine bacterium that has been the leading cause of foodborne outbreaks which leads to a significant threat to human health worldwide. Consumption of seafood contaminated with *V. parahaemolyticus* causes acute gastroenteritis in individuals. The bacterium poses two main virulence factor including the thermostable direct hemolysin (*tdh*) which is a pore-forming protein that contributes to the invasiveness of the bacterium in humans and TDH-related hemolysin (*trh*), which plays a similar role as *tdh* in the disease pathogenesis. This study aimed to investigate the antimicrobial resistance *V. parahaemolyticus* strains in shrimps purchased from wetmarkets and supermarkets. The *toxR*-based PCR assay indicated that a total of 57.8% (185/320) isolates were positive for *V. parahaemolyticus*. Only 10% (19/185) *toxR*-positive isolate exhibit the *trh* gene and none of the isolates were tested positive for *tdh*. The MAR index was measured for 14 common antimicrobial agents. The results indicated 98% of the isolates were highly susceptible to imipenem, ampicillin sulbactam (96%), chloramphenicol (95%), trimethoprim-sulfamethoxazole (93%), gentamicin (85%), levofloxacin (83%), and tetracycline (82%). The chloramphenicol (*catA2*) and kanamycin (*aphA-3*) resistance genes were detected in the resistant *V. parahaemolyticus* isolates. Our results demonstrate that shrimps are contaminated with *V. parahaemolyticus*, some of which carry the *trh*-gene thus being potential to cause food borne illness. The occurrence of multidrug resistance strains in the environment could be an indication of excessive usage of antibiotics in agriculture and aquaculture fields.

## INTRODUCTION

*Vibrio parahaemolyticus* is a Gram-negative halophilic, non-spore forming, curved rod-shaped bacterium that naturally lives in estuarine and marine environments worldwide (Su and Liu, 2007; Gode-Potratz et al., 2011; Ceccarelli et al., 2013; Zhang and Orth, 2013; Velazquez-Roman et al., 2014; Wu et al., 2014). While the majority of environmental strains are harmless members of the marine microbiota, some strains are opportunistic pathogens of humans (Johnson et al., 2008). Potential virulent *V. parahaemolyticus* strains are usually differentiated from likely avirulent strains by the presence of thermostable direct hemoylsin (*tdh*) and/or TDH related hemolysin (*trh*) genes (Bej et al., 1999). The *tdh* and *trh* genes are the two virulence factors associated with *V. parahaemolyticus* hemolysis and cytotoxicity activity in the host cell (Broberg et al., 2011; Zhang and Orth, 2013; Letchumanan et al., 2014). This halophile causes acute gastroenteritis in humans following the consumption of raw, undercooked or mishandled seafood (Zarei et al., 2012).

Although *V. parahaemolyticus* is often found in seafood, not all the strains are considered pathogenic (Velazquez-Roman et al., 2012). The strains isolated from environmental samples usually lack the pathogenic genes *tdh* and/or *trh* genes which cause illnesses to humans and marine animals (Deepanjali et al., 2005; Canizalez-Roman et al., 2011; Gutierrez West et al., 2013). Nevertheless, studies from US, Europe and Asia have reported around 0–6% of the environmental samples analyzed to be positive for the presences of *V. parahaemolyticus* strains with *tdh* gene and/or *trh* genes (DePaola et al., 2000; Vuddhakul et al., 2000; Wong et al., 2000; Alam et al., 2002; Hervio-Heath et al., 2002; Haley et al., 2014). In Malaysia, *V. parahaemolyticus* is naturally distributed in the marine coastal region of Malaysia. Its widespread incidence in the seawater allows the bacteria to use seafood as a vehicle of transmission and cause seafood borne gastroenteritis. *V. parahaemolyticus* is well known as one of the causative agent in regular institutional food poisoning cases in Malaysia (Al-Othrubi et al., 2014). Lately this bacterium has gain great attention from public due to the frequent rejection of seafood exported to EU countries (Abdul-Rahim et al., 2007; Al-Othrubi et al., 2014).

Conventional phenotyping and biochemical identification techniques of *V. parahaemolyticus* are often complicated when the strains are isolated from seafood and marine environments (Nishibuchi, 2006). These laborious protocols are mainly useful to estimate total load of *V. parahaemolyticus* in a sample as a potential risk estimation of the presence of pathogenic strains (Malcolm et al., 2015). Hence, this has raised concerns of many researchers, therefore suggesting the employ of molecular techniques to directly identify virulent markers (*tdh+* and *trh+*), since the pathogenic strains are cause of human illness. The polymerase chain reaction (PCR) assays is one of the molecular techniques that is widely used to detect the presences of pathogenic *V. parahaemolyticus* strain in food and environment (Panicker et al., 2004; Yamamoto et al., 2008; Paydar et al., 2013; Malcolm et al., 2015). PCR primers can be multiplexed in a single reaction to increase the detection limit or tailored as real-time PCR analysis to provide more rapid results (Grant et al., 2006; Zhang et al., 2014).

*Vibrio* spp. usually are said to be highly susceptible to most clinically used antibiotics (Mala et al., 2014; Shaw et al., 2014). However, over the years, antibiotic resistance strains has emerged into the environment due the excessive use of antibiotics and other chemotherapeutic agents in human, agriculture, and aquaculture fields (Cabello et al., 2013). In the aquaculture field, antimicrobials are used not to promote growth but rather to prevent (prophylactic use) and treat (therapeutic use) bacterial infections on fish and other invertebrates (Cabello et al., 2013). Oxytetracycline, tetracycline, quinolone, sulphonamides, and trimethoprim are among the antibiotics allowed and used in the Asian aquaculture industry to ensure continuous production of seafood (Rico et al., 2012; Yano et al., 2014). Antimicrobial resistant *V. parahaemolyticus* strains has been isolated and detected from shrimps in Thailand (Yano et al., 2014), Malaysia (Al-Othrubi et al., 2011; Sani et al., 2013), and China (Peng et al., 2010; Xu et al., 2014). This phenomenon has raised concern due to the increase number of resistance pathogenic *V. parahaemolyticus* strains in the environment toward clinically used antibiotics. There are many clinically used antibiotics as a choice of treatment for *Vibrio* spp. infections including cephalothin (first generation cephalosporins), cefuroxime (second generation cephalosporin), cefotaxime and ceftazidime (third generation cephalosporins), tetracycline, doxycycline, or fluoroquinolone (Tang et al., 2002; Al-Othrubi et al., 2014).

The use of antimicrobials in the aquaculture has caused the development of antibiotic resistant bacteria and antibiotic resistant genes. There are numerous antibiotic resistant genes can be found in bacteria and environments. For example, β-lactam and penicillin resistant genes *penA* and *blaTEM-1* (Srinivasan et al., 2005; Zhang et al., 2009), chloramphenicol resistant genes *cat*I, *cat*II, *cat*III, *cat*IV, and *floR* (Dang et al., 2007, 2008), tetracycline resistant genes *tatA, tatB, tatC, tatD, tatE, tatG, tatH, tatJ, tatY, tatZ*, and many more (Macauley et al., 2007; Zhang et al., 2009; Kim et al., 2013). These antibiotic resistant genes can be transfer among different bacteria via conjugation, transduction, or transformation (Manjusha and Sarita, 2011).

Plasmid is one of the important mediators that facilitate in the transfer of antibiotic resistant genes and it can be transmitted to the next generation via vertical gene transfer or exchanged with other bacteria via horizontal gene transfer (Okamoto et al., 2009; Manjusha and Sarita, 2011). Therefore, plasmids are cured in bacteria as a way to eliminate antibiotic resistance. There are various chemical and physical agents have been developed for plasmid curing. The conventional curing agents include chemical agents such as ethidium bromide (EB), acridine, and sodium dodecyl sulfate (SDS), and physical agents such as treatment with ultraviolet and growth at elevated temperature (Liu et al., 2012). A study conducted by Molina-Aja et al. (2002) reported that acridine orange and EB successfully cured the plasmids of Vibrios, whereas SDS did not cure any of the plasmid.

Shrimps are popular seafood with a high market demand in the aquaculture industry in South East Asia (Yano et al., 2014). Usually, shrimps are cooked prior to consumption though with the growing popularity of sushi, this crustacean is often eaten raw. The increase consumption of shrimps has encouraged the expansion of shrimp farming in many countries including Thailand (Yano et al., 2014), China (Xu et al., 2014), and Malaysia. The high contaminations of pathogenic *Vibrio* spp. in retail seafood in Malaysia recommend that there is a need for adequate consumer protection measures. Hence, monitoring the incidence of *V. parahaemolyticus* in Malaysian shrimp provides important information to consumers on the food safety. In this study, we evaluated the prevalence, antimicrobial susceptibility, and characterization of antibiotic resistant genes of *V. parahaemolyticus* in shrimps from retails in Malaysia.

## MATERIALS AND METHODS

### SAMPLING

The study mainly focused on two type of Malaysian shrimp, the banana prawn (*Penaeus indicus*), and red prawn (*Solenocera subnuda*). A total of 320 shrimp samples of both type were purchased from the local wetmarket and supermarket from January 2014–June 2014. All the samples were sealed and transported in an ice box to the laboratory for analysis on the same day.

### ENUMERATION AND ISOLATION OF *Vibrio* spp. IN SHRIMP SAMPLES

The enumeration and isolation of *V. parahaemolyticus* was carried out according to the method adapted from Zarei et al. (2012) with some minor modification. Twenty-five grams of samples were weighted and placed into a sterile homogenizer beg containing 225 mL of alkaline peptone water with 2% NaCl, pH 8.5, giving a first 10^-1^ dilution. The samples were homogenized for 60 s using a stomacher (Bagmixer 400W, Interscience, St Nom, France). Spread plate technique was employed in the enumeration of total presumptive *Vibrio* spp. in respective samples as described by Beneduce et al. (2010). Hundred microliter of each pre-enriched homogenates with appropriate sample dilution (1:10, 1:100, and 1:1000) were spread in duplicates onto the thiosulphate citrate bile salt sucrose (TCBS) agar (HiMedia, India) and incubated at 37^∘^C for 18–24 h. After incubation, the total colony count is determined and their concentrations in the original shrimp in cfu/mL were calculated.

For the isolation step, the remaining homogenate is incubated at 37^∘^C under aerobic conditions for 18 h. After 18 h of incubation and enrichment, a loopful of enriched mixture were streaked onto selective media, TCBS agar (HiMedia, India) and incubated at 37^∘^C for 18 h. The non-sucrose forming colony appeared green color on TCBS agar were picked and purified by streaking onto Tryptic Soy Agar (TSA; HiMedia, India) plates supplemented with 2% w/v sodium chloride (NaCl; Vivantis, USA). The TSA agar plates are incubated at 37^∘^C under aerobic conditions for 18–24 h. A loopful of pure isolate are inoculated into respective semi-solid nutrient agar and TSB with 30% glycerol, incubated at 37^∘^C for 18 h and then stored until further analysis.

### DNA EXTRACTION

The DNA from presumptive *Vibrio* spp. colonies was extracted using direct boiled cell lysate method (Suzita et al., 2010; Vengadesh et al., 2012). The colonies from semisolid nutrient agar are revived in Tryptic Soy Broth (TSB; HiMedia) with 2% w/v NaCl (Vivantis, USA) and incubated in a shaker incubator at 220 rpm for 37^∘^C for 18 h. Then, 1.5 mL of overnight culture suspension is pipette into a sterile 1.5 mL Eppendorf tube and centrifuged at 10,000 rpm for 5 min. The supernatant were carefully discarded and 1 ml of sterile ultrapure water were added, vortexed, and boiled at 100^∘^C for 7 min. After 7 min, the tubes are swiftly immerged onto ice for 5 min and then centrifuged at 13,000 rpm for 1 min to separate the debris and DNA contained supernatant. The supernatant were carefully pipetted and transferred into a new 1.5 mL microcentrifuge tube. The crude DNA was used as a template for PCR analysis.

### IDENTIFICATION OF *Vibrio parahaemolyticus* USING tox*R*-BASED PCR ASSAY

The *toxR*-based PCR assay was preformed to identify *V. parahaemolyticus* from all the presumptive isolates. Detection of *toxR* gene was carried out using primer *toxR*-F (5^′^-ATA CGA GTG GTT GCT GTC ATG-3^′^) and *toxR*-R (5^′^-GTC TTC TGA CGC AAT CGT TG-3^′^) with the expected amplicon size of 368 bp (Kim et al., 1999). The reaction mixture for this PCR assay was performed in a final volume of 20 μL, containing 2 μL of DNA template, 10 μL of 2x *Taq PLUS* PCR Smart mix 1 (SolGent^TM^, Korea), 6 μL of sterile distilled water and 1 μL of each primer. *toxR*-based PCR amplification were performed using PCR thermocycler (Kyratec, SuperCycler Thermal Cycler, Australia) with the following cycling conditions: initial denaturation at 95^∘^C for 4 min, 35 cycles of 94^∘^C for 1 min, 68^∘^C for 1 min and 72^∘^C for 30 s, and a final elongation at 72^∘^C for 5 min. PCR products were visualized by 1.5% agarose gel.

### DETECTION OF VIRULENCE GENE

The detection of *V. parahaemolyticus* virulence genes, *tdh* and *trh* was performed in a duplex PCR using specific primer adapted from Bej et al. (1999). PCRs were performed in a final volume of 20 μL, containing 2 μL of DNA template, 10 μL of 2x *Taq PLUS* PCR Smart mix 1 (SolGent^TM^, Korea), 4 μL of sterile distilled water and 1 μL of each primer. The PCR amplifications were performed using a Thermocycler (Kyratec, SuperCycler Thermal Cycler, Australia) with the following cycling conditions: initial denaturation at 94^∘^C for 3 min, 30 cycles of 94^∘^C for 1 min, 58^∘^C for 1 min and 72^∘^C for 1 min, and a final elongation at 72^∘^C for 5 min. All PCR products were visualized by 1.5% agarose gel.

### ANTIBIOTIC SUSCEPTIBILITY TEST

Fourteen antibiotic disks (Oxoid, UK) infused with amplicin (10 μg), ampicillin/sulbactam (30 μg), amikacin (30 μg), cefotaxime (30 μg), ceftazidime (30 μg), chloramphenicol (30 μg), gentamicin (30 μg), imipenem (10 μg), kanamycin (30 μg), levofloxacin (5 μg), nalidixic acid (30 μg), oxytetracycline (30 μg), sulfamethoxazole/trimethoprim (25 μg), and tetracycline (30 μg) were used in this study. The antibiotic susceptibility of *Vibrio* spp. isolates were studied using the disk diffusion method (Yano et al., 2014). The antibiotic disks were dispensed on Mueller Hilton agar (HiMedia, India) supplemented with 2% w/v NaCl (Vivantis, USA) plates with bacterial lawn. After incubation at 37^∘^C for 18 h, the inhibition zone is measured and interpreted guidelines of the Clinical and Laboratory Standard Institute (CLSI) (2010) M45-A2.

### PCR AMPLIFICATION OF ANTIBIOTIC RESISTANCE GENES AND PLASMID CURING

Isolates that showed an antimicrobial resistance phenotype were screened for the presence of genes coding for resistance determinants. The genes associated with resistance to B-lactams (*blaTEM, blaSHV, blaOXA*), tetracyclines (*tetA, tetB, tetC, tetG*), chloramphenicols (*catA1, catA2, catA3, catB3*), and kanamycin (*aphA-3*) were analyzed by PCR (Kim et al., 2013). PCR primers for each antibiotic gene were performed in a final volume of 20 μL, containing 2 μL of DNA template, 10 μL of 2x *Taq PLUS* PCR Smart mix 1 (SolGent^TM^, Korea), 6 μL of sterile distilled water and 1 μL of each primer. All primers are listed in **Table 1**. The PCR amplifications were performed in a Thermocycler (Kyratec, SuperCycler Thermal Cycler, Australia) with the following conditions: initial denaturation at 95^∘^C for 3 min, 35 cycles of 94^∘^C for 30 s, 52^∘^C for 1 min and 72^∘^C for 1.5 min, and a final elongation at 72^∘^C for 6 min. PCR products were visualized by 1.5% agarose gel electrophoresis. The isolates that exhibit presence of antibiotic resistant genes were subjected to plasmid curing.

**Table 1:** Polymerase chain reaction (PCR) primers targeting antibiotic resistant gene.

Antibiotics	Targetgene	Sequence 5^′^-3^′^	Amplicon size (bp)
Ampicillin	*blaSHV*	FW- TTATCTCCCTGTTAGCCACC RV- GATTTGCTGATTTCGCTCGG	796
	*blaOXA*	FW- ACCAGATTCAACTTTCAA RV- TCTTGGCTTTTATGCTTG	589
	*blaTEM*	FW- ATAAAATTCTTGAAGAC RV- TTACCAATGCTTAATCA	1073
Chloram phenicol	*catA1*	FW- CGCCTGATGAATGCTCATCCG RV- CCTGCCACTCATCGCAGTAC	456
	*catA2*	FW- ATGAATTTTACCAGAATTGATCTGAA RV- ATTTCAGTATGTTATCACACATCATCT	639
	*catA3*	FW- AAATTGGGTTCGCCGTGA RV- ATTTACTGTTACACAACTCTTGTAGCC	1863
	*catB3*	FW- TCAAAGGCAAGCTGCTTTCTGAGC RV- TATTAGACGAGCACAGCATGGGCA	566
Kanamycin	*aphA-3*	FW- GGGACCACCTATGATGTGGAACG RV- CAGGCTTGATCCCCAGTAAGTC	600
Tetracycline	*tetA*	FW- GTAATTCTGAGCACTGTCGC FV- CTGCCTGGACAACATTGCTT	956
	*tetB*	FW- ACGTTACTCGATGCCAT RV- AGCACTTGTCTCCTGTT	1169
	*tetC*	FW- AACAATGCGCTCATCGT RV- GGAGGCAGACAAGGTAT	1138
	*tetG*	FW- CCGGTCTTATGGGTGCTCTA RV- CCAGAAGAACGAAGCCAGTC	603

All antibiotic resistant isolates were subjected to a curing treatment using EB (Bio Basic, Canada). The isolates were grown in fresh TSB (HiMedia, India) and TSB supplemented with 0.2 mg/mL EB (Bio Basic, Canada), then incubated at 37^∘^C for 24 hours under constant agitation. After treatment with the curing agent, the profiles of resistance phenotypes and the related genes were examined for the antibiotic susceptibility profile and presence of resistance gene using PCR.

### STATISTICAL ANALYSIS

The experimental data was analyzed by using SPSS software version 20. Statistical analysis was performed in order to determine whether there were any significant difference in the species of crustaceans and the MAR index of resistant isolates using the independent *t*-test. The significance level was set at *p*-value of <0.05.

## RESULTS

### PREVALENCE OF *Vibrio parahaemolyticus* IN SHRIMP

*Vibrio parahaemolyticus* is a foodborne pathogen with a worldwide distribution but its densities in the environment and seafood vary depending on the season, location, sample type, and analytical methodology employed (Martinez-Urtaza et al., 2008; Zarei et al., 2012). In the present study, banana prawn (*P. indicus*; *n* = 160) and red prawn (*S. subnuda*; *n* = 160) samples were collected from three wetmarket and three supermarket. *V. parahaemolyticus* was found in all the shrimp samples using the conventional plating method. A total of 320 presumptive *V. parahaemolyticus* colonies that appeared green or bluish green on TCBS were picked. The results from the PCR showed positive amplification of *toxR* gene in 57.8% (185/320) of the *V. parahaemolyticus* isolates. Out of the 185 positive *V. parahaemolyticus* isolates, 52% (97/185) of the isolates are from red prawn (*S. subnuda*), and 48% (88/185) of the isolates are from banana prawn (*P. indicus*).

**Table 2** summarizes the total *Vibrio* densities (log cfu/mL) in shrimp. The banana prawn and red prawn collected from all the wetmarket and supermarket had a mean total *Vibrio* count range of 4.36 log cfu/mL to 6.34 log cfu/mL. Among the shrimp samples collected from wetmarket, the red prawn sample from wetmarket A had the highest mean total *Vibrio* counts of 6.34 log cfu/mL. The shrimps from wetmarket B had mean total *Vibrio* counts of 5.04 log cfu/mL, which is the lowest compared to the samples from wetmarket A and wetmarket C. Based on the results, the supermarket samples was least contaminated compared to wetmarket samples with mean total *Vibrio* counts of 4.35 log cfu/mL to 4.43 log cfu/mL.

**Table 2:** Total *Vibrio* densities (log cfu/mL) in shrimp.

Type of sample	Total *Vibrio* densities (log cfu/mL)					
	Wetmarket A	Wetmarket B	Wetmarket C	Supermarket A	Supermarket B	Supermarket C
Banana Prawn	6.24	5.04	5.16	4.36	4.40	4.21
Red Prawn	6.34	5.04	5.19	4.43	4.35	4.36

### DETECTION OF *tdh* and *trh*

To detect pathogenic isolates, *tdh* and *trh* genes were amplified using a duplex PCR. No *tdh*-positive *V. parahaemolyticus* isolates were detected among the 185 *toxR*-positive isolates. However, 19 (10%) *trh*-positive isolates were identified among the *V. parahaemolyticus* isolates. **Table 3** shows the *trh*-positive strains from the both shrimp species.

**Table 3:** List of *trh*-positive *Vibrio parahaemolyticus* isolates.

Strains	Shrimp species	Location	*toxR*-positive	*trh*-positive
VP89	Red Prawn	Supermarket C	+	+
VP90	Red prawn	Supermarket C	+	+
VP91	Red prawn	Supermarket C	+	+
VP92	Red prawn	Supermarket C	+	+
VP93	Banana prawn	Wetmarket A	+	+
VP94	Banana prawn	Wetmarket A	+	+
VP95	Banana prawn	Wetmarket A	+	+
VP96	Banana prawn	Wetmarket A	+	+
VP97	Banana prawn	Wetmarket A	+	+
VP98	Banana prawn	Wetmarket A	+	+
VP99	Banana prawn	Wetmarket A	+	+
VP100	Banana prawn	Wetmarket A	+	+
VP101	Banana prawn	Wetmarket A	+	+
VP102	Banana prawn	Wetmarket A	+	+
VP103	Banana prawn	Wetmarket A	+	+
VP175	Banana prawn	Supermarket C	+	+
VP176	Banana prawn	Supermarket C	+	+
VP177	Banana prawn	Supermarket C	+	+
VP178	Banana prawn	Supermarket C	+	+

### ANTIMICROBIAL SUSCEPTIBILITIES OF *Vibrio parahaemolyticus* ISOLATES

All 14 antibiotics used in this study are among the antibiotics recommended by Centre for Disease Control and Prevention (CDC) for the treatment of *Vibrio* spp. infections that includes fluoroquinolones (levofloxacin), cephalosporins (cefotaxime and ceftazidime), aminoglycosides (amikacin and gentamicin), and folate pathway inhibitors (trimethoprim-sulfamethoxazole; Daniels et al., 2000; Shaw et al., 2014). **Table 4** summarizes the percentage of antibiotic resistant of *V. parahaemolyticus* isolated from shrimp. The results indicated 82% of the isolates were resistant to ampicillin. Besides ampicillin, this study found that isolates exhibited resistant profile to aminoglycosides antimicrobial agents. A total of 95 isolates (51%) were resistant to amikacin, 28% resistant to kanamycin and 11% were gentamicin-resistant isolates. A high percentage of antibiotic resistant profile was also detected among the *V. parahaemolyticus* isolates toward the third generation cephalosporins (cefotaxime 37% and ceftazidime 15%). In the present study, high susceptibility to antibiotics including imipenem (98%), ampicillin sulbactam (96%), chloramphenicol (95%), trimethoprim-sulfamethoxazole (93%), gentamicin (85%), levofloxacin (83%), and tetracycline (82%) were observed among the *V. parahaemolyticus* isolates.

**Table 4:** The percentage of antibiotic resistant of *V. parahaemolyticus* isolated from shrimp.

Antimicrobial agent	No of Isolates (* %)					
	Red prawn		Banana prawn		Total isolates for each antimicrobial (** %)
	Wetmarket (*n* = 65)	Supermarket (*n* = 32)	Wetmarket (*n* = 37)	Supermarket (*n* = 51)	
Ampicillin	45 (69)	32 (100)	35 (95)	39 (76)	151 (82)
Ampicillin/sulbactam	0 (0)	0 (0)	0 (0)	3 (6)	3 (2)
Amikacin	20 (31)	17 (53)	22 (6)	36 (71)	95 (51)
Cefotaxime	13 (20)	12 (38)	9 (24)	35 (68)	69 (37)
Ceftazidime	0 (0)	1 (3)	2 (5)	25 (49)	28 (15)
Chloramphenicol	0 (0)	5 (16)	1 (3)	2 (4)	8 (4)
Gentamicin	0 (0)	1 (3)	0 (0)	20 (39)	21 (11)
Imipenem	0 (0)	1 (3)	1 (3)	2 (4)	4 (2)
Kanamycin	3 (5)	5 (16)	12 (32)	32 (63)	52 (28)
Levofloxacin	0 (0)	0 (0)	0 (0)	17 (33)	17 (9)
Nalidixic acid	0 (0)	0 (0)	0 (0)	35 (68)	35 (19)
Oxytetracycline	3 (5)	7 (22)	3 (8)	22 (43)	35 (19)
Sulfamethoxazole /trimethoprim	0 (0)	0 (0)	1 (3)	6 (12)	7 (4)
Tetracycline	3 (5)	3 (9)	5 (14)	20 (39)	31 (17)

( * % ) – No of isolates/Total isolates per source; (** %) – No of isolates/Total isolates.

In the current study, a high percentage (83%) of isolates have MAR index more than 0.2. The range of MAR index was from 0.00 to 0.79, with the highest MAR index attributed from an isolate (VP152; **Table 5**) from supermarket banana prawn which exhibited resistant to 11 antibiotics. Gwendelynne et al. (2005) stated that MAR indices higher that 0.2 could be due to contamination from high risk sources, thus leading to human health risk. A total of 49/185 isolates exhibited MAR index of 0.07, indicating the isolates were resistant to at least one type of antibiotic. About 28% of the isolates were resistant to three different antibiotics and have a MAR index of 0.21. The study also noted shrimp samples from wetmarket and supermarket had difference MAR indices.

**Table 5:** Antibiograms and multiple antimicrobial resistance (MAR) indices of *V. parahaemolyticus* strains.

Antibiograms	Strains	Total antibiotic resistance	MAR index
AK/AMP/CAZ/CN/CTX/K/NA/OT/SAM/SXT/TE	VP152	11	0.79
AK/AMP/CAZ/CN/CTX/K/LEV/ NA/OT/TE	VP134, VP135, VP139	10	0.71
AK/AMP/CAZ/CN/CTX/K/NA/OT/SXT/TE	VP158	10	0.71
AK/CAZ/CN/CTX/K/LEV/NA/OT/TE	VP136	9	0.64
AK/C/CAZ/CN/CTX/K/NA/OT/TE	VP160	9	0.64
AK/AMP/CAZ/CN/CTX/K/NA/ SAM	VP142	8	0.57
AK/AMP/CAZ/CTX/CN/K/OT/TE	VP153	8	0.57
AK/AMP/CAZ/CN/CTX/K/LEV/ NA	VP165	8	0.57
AK/CAZ/CN/CTX/K/LEV/NA/SXT	VP174	8	0.57
AK/CAZ/CN/CTX/K/NA/OT/TE	VP162	8	0.57
AK/AMP/C/CAZ/CN/K/NA/ SXT	VP158	8	0.57
AK/AMP/K/LEV/NA/OT/TE	VP130	7	0.5
AK/AMP/CAZ/CN/CTX/K/NA	VP163	7	0.5
AK/AMP/CTX/LEV/NA/OT/TE	VP137, VP167	7	0.5
AK/AMP/CAZ/CN/K/NA/OT	VP138	7	0.5
AK/AMP/CTX/K/IMP/OT	VP71	6	0.43
AK/CAZ/CN/CTX/K/NA	VP132, VP133, VP140, VP141, VP143	6	0.43
AMP/IPM/LEV/NA/OT/TE	VP145,VP146	6	0.43
AK/AMP/CAZ/CTX/K/NA	VP151	6	0.43
AK/AMP/CAZ/CTX/K/SAM	VP157	6	0.43
AK/CAZ/CTX/K/NA/SXT	VP161	6	0.43
AK/AMP/CTX/K/LEV/NA	VP166, VP169	6	0.43
AMP/CTX/NA/OT/SXT/TE	VP170	6	0.43
AK/AMP/CAZ/CTX/K	VP102, VP103, VP148	5	0.36
AMP/LEV/NA/OT/TE	VP131	5	0.36
CAZ/CTX/K/NA/OT	VP168	5	0.36
AMP/CTX/NA/OT/TE	VP173	5	0.36
AK/AMP/C/OT/TE	VP183	5	0.36
AMP/CTX/OT/TE	VP159	4	0.29
AK/AMP/CAZ/CTX	VP90	4	0.29
AK/AMP/K/SXT	VP119	4	0.29
AK/AMP/OT/TE	VP125	4	0.29
AMP/NA/OT/TE	VP129	4	0.29
LEV/NA/OT/TE	VP144	4	0.29
AK/AMP/LEV/NA	VP171	4	0.29
AK/AMP/CTX/K	VP73, VP77, VP82, VP95, VP104, VP117, VP149, VP154, VP156	4	0.29
AK/AMP/K/CN	VP84	4	0.29
AK/AMP/CTX	VP21, VP30, VP52, VP54, VP64, VP70, VP78, VP87, VP88, VP89, VP91, VP111, VP126	3	0.21
AK/AMP/K	VP55, VP93, VP101, VP110, VP118, VP123, VP128, VP150	3	0.21
AMP/OT/TE	VP59, VP72, VP74, VP108, VP120	3	0.21
AMP/CTX/K	VP43	3	0.21
AK/AMP/IMP	VP114	3	0.21
AMP/C/CTX	VP182	3	0.21
AMP/C/OT	VP179, VP180, VP181	3	0.21
AMP/LEV/NA	VP172	3	0.21
C/OT/TE	VP184	3	0.21
AMP/TE	VP2, VP105	2	0.14
OT/TE	VP5	2	0.14
AMP/K	VP12	2	0.14
AMP/CTX	VP28, VP29, VP46, VP50, VP99, VP127, VP147, VP175,	2	0.14
AK/AMP	VP31, VP34, VP41, VP45, VP46, VP57, VP60, VP61, VP63, VP66, VP79, VP83, VP85, VP107, VP109, VP112, VP113, VP121, VP124, VP178	2	0.14
AK/CTX	VP42, VP44	2	0.14
AMP	VP6, VP7, VP8, VP9, VP10, VP11, VP16, VP18, VP19, VP20, VP22, VP25, VP27, VP32, VP33, VP35, VP37, VP38, VP48, VP51, VP53, VP58, VP62, VP67, VP68, VP69, VP75, VP76, VP80, VP81, VP86, VP92, VP94, VP96, VP97, VP100, VP106, VP115, VP116, VP122, VP155, VP164, VP176, VP177	1	0.07
AK	VP47, VP49	1	0.07
CTX	VP23, VP26, VP40	1	0.07

AMP, Amplicin; OT, Oxytetracycline; NA, Nalidixic acid; C, Chloramphenicol; CTX, Cefotaxime; SXT, Suphamethox/Trithoprim; IMP, Imipenem; AK, Amikacin; SAM, Ampicillin/Sulbactam; LEV, Levofloxacin; CAZ, Ceftazidime; K, Kanamycin; CN, Gentamicin; TE, Tetracycline.

**FIGURE 1:**
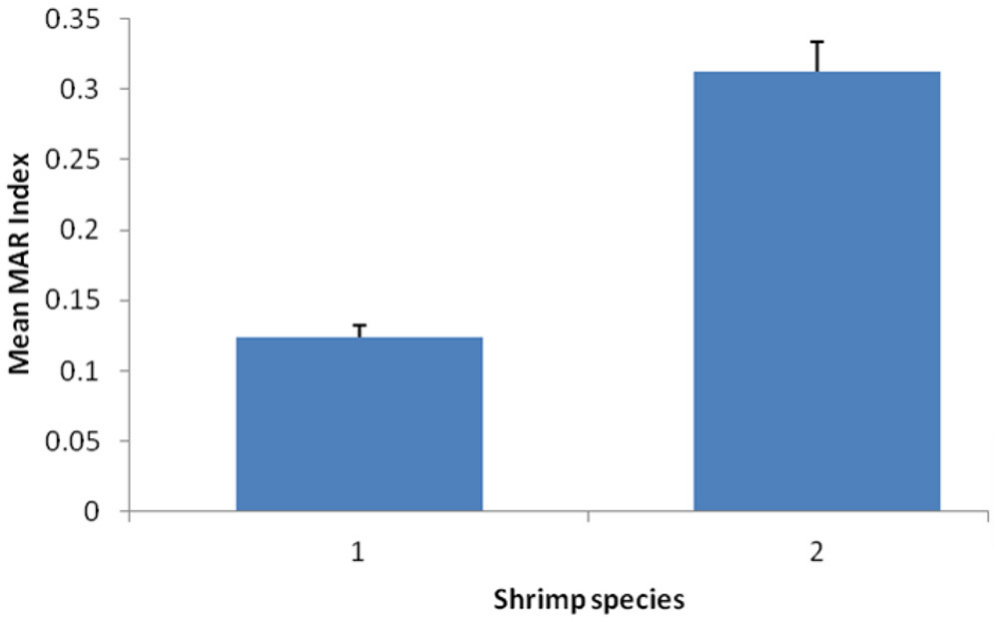
**Comparison of the mean of MAR index for *Vibrio parahaemolyticus* isolates of different shrimp species.** Each bar represents the mean of the MAR index for *V. parahaemolyticus* strains from different shrimp species. Label 1, red prawn; Label 2, banana prawn.

Furthermore, the mean of MAR index for *V. parahaemolyticus* isolates of different shrimp species were compared and the results showed there was significantly difference with *p* < 0.05 (**Figure 1**). The mean MAR index of the *V. parahaemolyticus* isolates recovered from banana prawn species were significantly higher than mean MAR index of *V. parahaemolyticus* isolates from the red prawn species. Results in this study highlighted that the banana prawn isolates are possible exposed to various antimicrobials that lead to emerging of multiple antibiotic resistant strains.

### DETECTION OF ANTIMICROBIAL RESISTANCE GENE AND PLASMID CURING

Plasmid curing was performed to detect the location of antibiotic resistance genes in the resistant *V. parahaemolyticus* isolates. All the chloramphenicol-resistant *V. parahaemolyticus* isolates (*n* = 8) were screened for chloramphenicol *catA1, catA2, catA3*, and *catB3* genes. Only *catA2* gene was detected and present in all the eight chloramphenicol-resistant *V. parahaem*olyticus isolates. Two *V. parahaemolyticus* isolates (VP160 and VP185) from banana prawn Supermarket B lost their resistance toward chloramphenicol as a result of plasmid curing. The PCR result showed *catA2* gene was not present after plasmid curing suggesting the gene is located in the plasmid of the isolate. The plasmid curing result for another six chloramphenicol-resistant *V. parahaemolyticus* isolates (VP179, VP180, VP181, VP182, VP183, VP184) showed possibility that is chromosomal-mediated since the isolates exhibit positive amplification with *catA2* gene and demonstrated phenotypic resistance toward chloramphenicol on the disk diffusion test after plasmid curing.

The kanamycin *aphA-3* gene was detected in 15 out of 52 kanamycin-resistant *V. parahaemolyticus isolates* (VP84, VP133, VP134, VP136, VP138, VP140, VP142, VP143, VP151, VP153, VP160, VP161, VP162, VP163, VP174). This study did not detect any β-lactam resistance genes (*blaSHV, blaOXA*, and *blaTEM*) which are normally found as plasmid-encoded β-lactamase and tetracycline resistant genes (*tetA, tetB, tetC*, and *tetG*) among the *V. parahaemolyticus* isolates.

## DISCUSSION

The presence of *V. parahaemolyticus* in shrimp samples suggests a public health concern to those who consume shrimp raw or undercooked shrimps. The risk is substantial regardless of pathogenicity island presence in the genomes of circulating *V. parahaemolyticus*, since some illnesses are caused by isolates lacking of *tdh*, *trh*, or T3SS2 (García et al., 2009; Harth et al., 2009; Chao et al., 2010; Haley et al., 2014). A total of 185 isolates recovered in this study were *toxR*-positive with a mean total *Vibrio* count range of 4.36 log cfu/mL to 6.34 log cfu/mL. Results of this study demonstrated a high level of *V. parahaemolyticus* contamination in shrimp samples from wetmarket compared to supermarket samples.

Since there is limited access to the main source origin of the samples, we scrutinized the environment at the site whereby samples are collected. The occurrence of *Vibrio* spp. in both shrimp species samples are probably a reflection of the atmosphere at the wetmarket and supermarket. The contamination occurs due to way of handling shrimps by wetmarket retailers is less hygienic compared to supermarkets retailers. The wetmarket retailers display the shrimps on ice rack left on the normal room temperature which allows the ice to melt faster. Where else, at the supermarket, retailers display their shrimps on ice in air conditional room temperature. The difference of temperature in the environment contributes to the high counts of *V. parahaemolyticus* in wetmarket as seen in our study. Previous study has indicated that seafood such as fish should be kept in cold condition during transit and storage to reduce the risk and level of Vibrios (Elhadi et al., 2004). Sudha et al. (2014) have also reported higher contamination of shellfishes with pathogenic Vibrios isolated from samples collected from the roadside stalls compared to markets in Cochin, India.

The study results are in line with previous studies that stated the occurrence of *V. parahaemolyticus* in supermarket samples is possibly due to lack of hygiene, improper handling, cross contamination, or difference in storage temperature during the capture to the supermarket (Yang et al., 2008; Tunung et al., 2010; Sudha et al., 2014). *V. parahaemolyticus* cells multiple rapidly with increase in ambient temperature, hence non-refrigerated post harvest storage could possible lead consumers to the exposure of potentially pathogenic *V. parahaemolyticus* strains (Sudha et al., 2014). This pathogen cell viability could be reduced if the samples are maintained on ice (Su and Liu, 2007). Therefore, in effort to reduce the risk of *V. parahaemolyticus* in seafood, retailers from wetmarket, and supermarket should be well educated on seafood handling techniques, storage temperature conditions, and proper hygiene.

Seafood is known as a vehicle of transmission of food borne bacteria. The virulence of these bacteria plays an important role in causing human illness. In order to assess the real risk factor of *V. parahaemolyticus* in the food sample, their identification should be followed with the detection of the virulence genes. Only 10% (19/185) of *V. parahaemolyticus* isolates in the study was *trh*-positive and none was *tdh*-positive. These results are not surprising since it is reported that environmental *V. parahemolyticus* strains are considered to be non pathogenic due to lacking *tdh* or *trh*, but a small percentage of these environmental strains harbor either one or both of the virulence factors (Wong et al., 2000; Alam et al., 2002; Hervio-Heath et al., 2002; Velazquez-Roman et al., 2012; Haley et al., 2014).

The *trh* gene is considered as a virulence factor of *V. parahaemolyticus* and plays a similar role as *tdh* gene in the pathogenesis of *V. parahaemolyticus* (Nelapati et al., 2012). The study findings are in agreement with another study that reported the detection of 12% (6/50) isolates to harbor *trh* gene in the food samples in Malaysia (Paydar et al., 2013). A recent study reported high occurrence of *tdh+* and *trh+* isolates in shrimp and cockles in Malaysia. In that study, 26 isolates were positive for *trh* virulence gene and only eight isolates positive for *tdh* virulence gene isolated from cockles and shrimp (Al-Othrubi et al., 2014). The variation in the occurrence of pathogenic *V. parahaemolyticus* between the current study and previous study may be associated with differences in sampling techniques, sample sources, and the detection techniques employed. Wilson and Salyers (2003) stated that environmental factor including interaction with other hosts plays a huge effect in the evolution of certain pathogens.

The study results demonstrated 82% of the isolates were resistance to ampicillin. It is in agreement with other studies that reported *V. parahaemolyticus* isolated from seafood samples are commonly resistance to ampicillin (Okuda et al., 1997; Han et al., 2007; Al-Othrubi et al., 2014). The ampicillin-resistant pattern could be due to the fact that first generation antibiotics including ampicillin is misused in the environment thus reducing the susceptibility and low efficiency of ampicillin in treatment of *Vibrio* infection (Sudha et al., 2014). Furthermore, high percentage of antibiotic resistance profile was also detected among the *V. parahaemolyticus* isolates toward the third generation cephalosporins (cefotaxime 37% and ceftazidime 15%). The results are in agreement with Sahilah et al. (2014) that the presence of cefuroxime (second generation cephalosporin) and ceftazidime-resistant *V. parahaemolyticus* isolates were evidenced in shellfish samples from Terengganu, Malaysia. A study in Korea also demonstrated high percentage (70–80%) of the *V. parahaemolyticus* isolates from seafood are resistant to both cefotaxime and ceftazidime (Jun et al., 2012). In contrast, a study in U.S. reported low percentage (3%) of *V. parahaemolyticus* isolates are resistant to cefotaxime (Shaw et al., 2014). The discrepancies in the literature regarding the susceptibility of *V. parahaemolyticus* to cefotaxime could be related to test methodology or geographical variation of samples.

In the present study, high susceptibility to antibiotics including imipenem (98%), ampicillin sulbactam (96%), chloramphenicol (95%), trimethoprim-sulfamethoxazole (93%), gentamicin (85%), levofloxacin (83%), and tetracycline (82%) were observed among the *V. parahaemolyticus* isolates. These findings were in agreement with literature stating most *V. parahaemolyticus* isolates were susceptible to chloramphenicol and tetracycline (Han et al., 2007; Sahilah et al., 2014; Sudha et al., 2014). *V. parahaemolyticus* strains isolated from fish were also reported to be highly susceptible to imipenem (Noorlis et al., 2011). Based on these findings, besides the widely use tetracycline, imipenem could be prescribed by doctors as a treatment for bacterial infection.

The shrimp collected from wetmarket and supermarket had different MAR indices with a range from 0.00 to 0.79. The highest MAR index attributed from an isolate (VP152) from supermarket banana prawn which exhibited resistance to 11 antibiotics. Gwendelynne et al. (2005) stated that MAR indices higher that 0.2 could be due to contamination from high risk sources, thus leading to human health risk. The findings were in agreement with researchers in Malaysia which reported that the antimicrobial susceptibility of *V. parahaemolyticus* varied and influenced the resistance level depending on the source of sample obtained (Khan et al., 2007; Tunung et al., 2012) and the differences in the geographical location (Lesley et al., 2011). Moreover, this was further supported by the findings in this study showing that the mean MAR index of *V. parahaemolyticus* isolates from the two shrimps species varied significantly with *p* < 0.05. A higher mean MAR index was observed in the *V. parahaemolyticus* isolates from banana prawn collected from both wetmarket and supermarket. The banana prawn is more exposed to various antimicrobial in the environment compared to the red prawn. The high occurrence of multiple antibiotic resistance strains in this study could be due to intense usage of antibiotics to fight against bacterial infections in the aquaculture sector and maintaining a continuous production and supply of shrimp. A study in Thailand have highlighted that shrimp farming in the inland environments might increase the opportunity for dissemination of resistance genes among bacteria (Yano et al., 2014). Literature have also stated resistant *V. parahaemolyticus* strains could be isolated from samples collected from location with frequent usage of antibiotics. This is due to mutation that has modified the target site or transport mechanism which causes the antibiotics to become inactive on cell (Zulkifli et al., 2009). Such widespread usage of antimicrobial has increased antibiotic resistance among environmental bacteria including potential *Vibrio* spp. (Tendencia and de la Peña, 2001; Yano et al., 2014).

Chloramphenicol (*catA2*) gene was detected in eight chloramphenicol-resistance *V. parahaemolyticus* isolates. Two of the isolates had the gene present in their plasmid where else another six isolates showed possibility of chromosomal-mediated since the isolates exhibit positive amplification with *catA2* gene and demonstrated phenotypic resistance to chloramphenicol on the disk diffusion test after plasmid curing. These results are in agreement with previous study stating the presence of *V. parahaemolyticus* isolates that demonstrated chromosomal-mediated resistance against chloramphenicol (Devi et al., 2009). Researchers have also reported the presence of antibiotic resistance gene located in the chromosome of *Vibrio* spp. (Son et al., 1998; Manjusha and Sarita, 2011). The result of plasmid curing revealed that kanamycin-resistant *V. parahaemolyticus* isolates were potentially chromosomal-mediated since the isolates exhibit positive amplification with *aphA-3* gene and demonstrated phenotypic resistance or intermediate to kanamycin on the disk diffusion test after plasmid curing.

This study did not detect any β-lactam resistance genes (*blaSHV, blaOXA*, and *blaTEM*) which are normally found as plasmid-encoded β-lactamase and tetracycline resistance (*tetA, tetB, tetC*, and *tetG*) gene among the *V. parahaemolyticus* isolates. It is well known that the ampicillin-resistance genes are very diverse. Therefore, the negative results of all the tested β-lactamase genes and tetracycline genes could possibly due to possession of other encoding genes in all the ampicillin-resistant or tetracycline resistance *V. parahaemolyticus* isolates of the present study. For instance, a class A extended-spectrum- β-lactamase gene, *bla_PER-1_*, which is mostly associated with Gram-negative clinical pathogens such as *Pseudomonas aeruginosa* (Qing et al., 2014) was also detected in *V. parahaemolyticus* (Wong et al., 2012; Liu et al., 2013). Another literature has also stated that ampicillin resistance of *V. parahaemolyticus* was not conferred by the *bla* gene but was mediated by an eﬄux system (Pazhani et al., 2014). A study also has reported the occurrence of tetracycline genes *tetM* and *tetS* in *Vibrio* spp. from seawater in Japan and Korea which could be an important reservoir of tetracycline resistance genes in the marine environment (Kim et al., 2004).

To the best of our knowledge, our findings represents the first comprehensive report about the prevalence, antibiotic resistance profile, antibiotic resistance genes detection, and plasmid curing of *V. parahaemolyticus* isolates from shrimps in Malaysia. Most gastroenteritis cases are attributed by consumption of seafood including shrimps. Shrimps may act as a vehicle to disseminate potential pathogens to the consumers. The occurrence of pathogenic *V. parahaemolyticus* in banana prawn (*P. indicus*) and red prawn (*S. subnuda*) in this study requires extended surveillance in the region and across the country. Hence, continuous monitoring of *V. parahaemolyticus* strains and their susceptibility to antibiotics is necessary to ensure the best treatment for patients with gastroenteritis and ensure seafood safety.

## Conflict of Interest Statement

The authors declare that the research was conducted in the absence of any commercial or financial relationships that could be construed as a potential conflict of interest.
